# The Anticancer Effect of Genistein Through Enhancing PERK Signaling and Suppressing the IRE1α-XBP1 Axis in Canine Mammary Gland Tumor Cells

**DOI:** 10.3390/ani15121717

**Published:** 2025-06-10

**Authors:** Ye-Ji Jang, Min-Jae Yoo, Hyuk Jang, Jun Song, Sang-Youel Park, Jawun Choi, Jae-Won Seol

**Affiliations:** College of Veterinary Medicine, Jeonbuk National University, Iksan 54596, Republic of Korea; yejiown25@gmail.com (Y.-J.J.); ymin105@naver.com (M.-J.Y.); hjang1003@naver.com (H.J.); junsong93@naver.com (J.S.); sypark@jbnu.ac.kr (S.-Y.P.)

**Keywords:** canine mammary gland tumors, CMT-U27, genistein, unfolded protein response, estrogen receptor alpha

## Abstract

Genistein, a natural isoflavone, exerts anticancer effects on human breast cancer cells by modulating the unfolded protein response (UPR). However, the effect of genistein on UPR in canine mammary gland tumor (CMT) cells remains unknown. The aim of the present study was to investigate the anticancer effects of genistein on CMT-U27 cells, focusing on the regulation of UPR-related pathways and the associated cell death mechanisms. CMT-U27 cells were treated with genistein. Cell viability, apoptosis, and UPR-related protein expression were analyzed using MTS assay, Annexin V-Propidium Iodide (PI) staining, Western blotting, and immunocytochemistry. Genistein treatment significantly reduced cell viability and induced apoptosis, accompanied by an increased Bcl-2-associated X (Bax) ratio of B-cell lymphoma-2 (Bcl-2) and cleaved caspase-8 and caspase-3. On regulation of the UPR system, genistein treatment showed a dual-function by enhancing the protein kinase R-like endoplasmic reticulum kinase (PERK) signaling while suppressing the inositol-requiring enzyme 1 alpha (IRE1α)–X-box-binding protein 1 (XBP1) axis. Furthermore, genistein downregulated estrogen receptor alpha (ERα), which may contribute to the inhibition of IRE1α signaling through a disrupted positive feedback loop. These findings suggested that genistein modulates the UPR to induce apoptosis in CMT-U27 cells, highlighting its potential as a therapeutic or adjuvant agent for CMTs.

## Simple Summary

Breast cancer is one of the most common tumors in female dogs, and more than half of these cases are malignant. While surgery is the main treatment, it may not be effective when the cancer spreads or returns. In this study, we tested genistein, a natural compound found in soybeans, to see if it could help fight this type of cancer. We treated dog breast cancer cells in the laboratory and found that genistein slowed their growth and caused them to die. This effect was linked to changes in how the cells handle stress inside their structure. Genistein triggered signals that lead to cell death and blocked signals that help cancer cells survive. It also reduced the level of a hormone-related protein that is known to support tumor growth. These findings were confirmed using several laboratory methods. Our results suggest that genistein may help control the growth of breast cancer in dogs by affecting multiple biological pathways. This study provides early evidence that natural substances like genistein could become part of improved treatment options in veterinary medicine.

## 1. Introduction

Canine mammary gland tumors (CMTs) are the most frequently diagnosed neoplasms in intact female dogs, accounting for approximately 42% of all tumors, with over half (52.4%) classified as malignant [1,2]. Surgical excision remains the standard treatment, particularly for benign tumors and early-stage localized malignancies [3]. However, surgery alone offers limited therapeutic benefits in cases where distant metastasis is detected or the risk of dissemination is elevated. Even after mastectomy, the recurrence and incidence of metastases remain substantial, with reported rates as high as 26.5% [4]. Therefore, chemotherapy is administered as an adjuvant treatment, either preoperatively or postoperatively, in such cases.

Current chemotherapeutic strategies for cancer treatment primarily rely on cytotoxic agents, such as carboplatin [5], and tamoxifen [6], which are often administered in combination [7,8]. Nonetheless, the long-term efficacy of these conventional regimens is frequently impaired by issues such as dose-limiting toxicity [9] and the emergence of chemoresistance [10]. Therefore, there is increasing demand for novel therapeutic approaches to circumvent the limitations of standard chemotherapies. Among these alternatives, natural product-based therapeutics, including phytochemicals and herbal compounds, have gained attention because of their diverse anticancer mechanisms [11]. Furthermore, a growing body of evidence supports the therapeutic potential of combining natural compounds with conventional chemotherapeutic agents, improving treatment efficacy and attenuating drug resistance in various malignancies [12,13]. However, the potential use of natural products as adjunct treatments for CMTs remains largely unexplored.

Breast cancer cells are continually exposed to diverse microenvironmental stressors such as hypoxia, nutrient deprivation, and oxidative stress [14]. In addition, their elevated metabolic demands and accelerated proliferation impose a substantial burden on the protein-folding capacity of the endoplasmic reticulum (ER), resulting in ER stress. ER stress is characterized by the disruption of ER homeostasis, which is commonly triggered by disturbances in calcium signaling and the accumulation of misfolded or unfolded proteins. Under physiological conditions, the ER transmembrane sensors protein kinase R-like ER kinase (PERK), inositol-requiring enzyme 1 alpha (IRE1α), and activating transcription factor 6 (ATF6) are maintained in an inactive state through binding with the chaperone glucose-regulated protein 78 (GRP78, also known as BiP). However, GRP78 dissociates and initiates the unfolded protein response (UPR) signaling cascade upon stress induction [15]. The UPR has been widely implicated in promoting cancer cell survival, therapeutic resistance, and metastatic progression in breast cancer [16]. Notably, UPR activation is associated with resistance to commonly used chemotherapeutic agents such as paclitaxel [17] and cisplatin [18]. In the IRE1α–X-box binding protein 1 (XBP1) axis, knockdown of XBP1 has been shown to inhibit the growth of estrogen receptor 1 (ESR1)-positive breast cancer cells, demonstrating its pro-survival role in hormone-responsive tumors [19]. The activation of the PERK pathway leads to the upregulation of activating transcription factor 4 (ATF4), a transcription factor involved in amino acid metabolism [20], while elevated C/EBP homologous protein (CHOP) expression has been associated with enhanced cell death in certain breast cancer subtypes [21]. Although direct evidence implicating ATF6 in breast cancer progression is limited, indirect regulation of GRP78 and spliced XBP1 (XBP1s) has been reported in hepatocellular carcinoma, suggesting a potential mechanistic crosstalk among the UPR branches [22]. Consequently, growing interest has emerged in targeting components of the UPR as a therapeutic strategy to overcome the limitations of conventional breast cancer therapies.

Genistein, a predominant isoflavone derived from soybeans, was first isolated in 1899 from *Genista tinctoria* (Dyer’s broom), the plant species from which its nomenclature originated [23]. This compound exhibits therapeutic properties, including antioxidant, anti-inflammatory, and antiproliferative effects, in several diseases such as cancer and cardiovascular conditions [24]. It has demonstrated antineoplastic effects against multiple cancer types, including breast [25], prostate [26], and pancreatic cancers [27], primarily by activating mechanisms involving cell cycle arrest, apoptosis induction, and suppression of angiogenesis and metastasis. Emerging evidence has shown that genistein induces ER stress and modulates the UPR signaling pathway by upregulating GRP78, CHOP, calpain, and caspase-12, leading to apoptotic cell death [28]. Notably, the UPR pathway critically facilitates tumor cell adaptation to microenvironmental stress, resulting in the development of resistance to conventional chemotherapeutics. Therefore, targeting the UPR using genistein has emerged as a promising strategy for enhancing chemosensitivity and circumventing drug resistance to typical chemotherapy.

Although UPR has been extensively studied as a therapeutic target in human breast cancer, its functional role and clinical relevance in CMTs remain largely unexplored. Considering the critical involvement of the UPR in tumor cell survival and adaptation under stress conditions, investigating its therapeutic potential in CMTs is both timely and necessary. Accordingly, the aim of this study was to address this gap by exploring the anticancer effects of UPR modulation in CMT cells.

The aim of the present study was to investigate the anticancer effects of genistein on canine mammary gland tumor cells and elucidate its underlying mechanisms through UPR pathway modulation. We hypothesized that genistein exerts anti-tumor effects in CMT cells through UPR pathway modulation and the downregulation of estrogen receptor alpha (ERα). We performed in vitro assays, including cell viability analysis, Western blotting, and immunofluorescence, using CMT-U27 cells to test this hypothesis. Our findings demonstrated that genistein exerts its anticancer effects on CMT cells by modulating key components of the UPR pathway, thereby supporting its potential as a therapeutic candidate for CMTs.

## 2. Materials and Methods

### 2.1. Cell Culture and Reagents

We purchased CMT-U27 cells from the American Type Culture Collection (Manassas, VA, USA) and cultured them in Roswell Park Memorial Institute 1640 medium (CMT-U27 cells; HyClone, Logan, UT, USA) with 10% fetal bovine serum (FBS; Atlas Biologicals, Fort Collins, CO, USA), penicillin 100 unit/mL and streptomycin 100 μg (Sigma-Aldrich, St. Louis, MO, USA). Then, we incubated cells at 37 °C in 5% CO_2_. Genistein (≥98% purity, Sigma-Aldrich, G6649) was dissolved in dimethyl sulfoxide (DMSO) to prepare a 100 mM stock solution. For cell treatment, the stock was diluted at a ratio of 1 μL per 1 mL of complete medium, resulting in a final working concentration of up to 100 μM.

### 2.2. 3-(4,5-Dimethylthiazol-2-yl)-5-(3-carboxymethoxyphenyl)-2-(4-sulfophenyl)-2H-tetrazolium (MTS) Assay

CMT-U27 cells were seeded at a density of 1 × 10^4^ cells per well in 96-well plates containing 100 μL of medium and incubated at 37 °C for 24 h to assess cell viability using the 3-(4,5-dimethylthiazol-2-yl)-5-(3-carboxymethoxyphenyl)-2-(4-sulfophenyl)-2H-tetrazolium (MTS) assay. We treated cells with genistein at the indicated concentration for 24 h. CellTiter 96 AQueous One Solution Reagent 20 μL was added to each well and incubated for 2 h at 37 °C. The absorbance was measured at 490 nm using a microplate reader (Spectramax M2; Molecular Devices, San Jose, CA, USA).

### 2.3. Annexin v/Propidium Iodide (PI) Staining

Cell death of CMT-U27 was evaluated by flow cytometry using an Annexin V assay (Santa Cruz Biotechnology, Inc., Dallas, TX, USA) according to the manufacturer’s protocol. Annexin V content was estimated by measuring the fluorescence at 488 nm (excitation) and 525 nm (emission) using the Guava easyCyte HT system (Millipore, Billerica, MA, USA).

### 2.4. Western Blotting

We lysed CMT-U27 cells in a cold lysis buffer with a protease inhibitor cocktail (Sigma-Aldrich). Then, we extracted protein from lysed cells using sodium dodecyl sulfate–polyacrylamide gel electrophoresis and the gel was subsequently transferred to nitrocellulose membranes. The membranes were treated with 5% skimmed milk to inhibit non-specific binding. We incubated the membranes overnight at 4 °C with the following primary antibodies in a blocking buffer: rabbit polyclonal phosphorylated PERK (p-PERK) antibody (PA5-40294; Invitrogen, Waltham, MA, USA), rabbit polyclonal ATF4 antibody (BS-1531R; Bioss, Woburn, MA, USA), mouse monoclonal CHOP antibody (2895; Cell Signaling, Danvers, MA, USA), rabbit polyclonal death receptor 5 (DR5) antibody (ab8416; Abcam, Cambridge, UK), rabbit polyclonal microtubule-associated protein 1 light chain 3 beta (LC3B) antibody (4108; Cell Signaling), mouse monoclonal anti-B-cell lymphoma-2 (Bcl-2) antibody (Sc-7382; Santacruz Biotechnology, Dallas, TX, USA), rabbit polyclonal anti-Bcl-2-associated X (Bax) antibody (2772; Cell Signaling), rabbit polyclonal anti-Caspase-3 antibody (9662; Cell Signaling), mouse monoclonal anti-Caspase-8 antibody (551242; BD, Franklin Lakes, NJ, USA), rabbit polyclonal phosphorylated IRE1α (p-IRE1α) antibody (Ser724; Invitrogen), mouse monoclonal XBP1s antibody (27901; Cell Signaling), rabbit polyclonal GRP78 antibody (ab21685; Abcam), mouse monoclonal anti-ERα antibody (MA1-12692; Invitrogen), and mouse monoclonal anti-β-actin (A5441; Sigma-Aldrich). After that, we incubated the membranes with horseradish peroxidase (HRP)-conjugated secondary antibodies for 1 h at room temperature. We amplified chemiluminescent signals using WESTSAVE Gold (LF-QC0103; Abfrontier, Seoul, Republic of Korea) or WESTSAVE Star (LF-QC0106; Abfrontier). Using a Fusion FX7 acquisition system (Vilbert Lourmat, Eberhardzell, Germany), we captured the signals on the membranes. Quantity One (version 4.6.6) was used to quantify the band densities and normalize each density to β-actin. The densities were presented relative to that of the control.

### 2.5. Immunocytochemistry

We cultured CMT-U27 cells on gelatin-coated coverslips. Cells were fixed with 2% paraformaldehyde for 20 min at 4 °C and permeabilized using 0.5% Triton X-100 in phosphate-buffered saline (PBS) for 10 min. We performed the blocking procedure using 5% animal serum (donkey or goat) in 2% bovine serum albumin (BSA) in PBS for 1 h at room temperature. Then, we treated the cells with primary antibodies against p-IRE1 alpha (Invitrogen), XBP1s (Cell Signaling), p-PERK (Invitrogen), GRP78 (Abcam), ATF4 (Bioss), CHOP (Cell Signaling), active caspase-3 (R&D Systems, Minneapolis, MN, USA) and ERα (Invitrogen) in the blocking solution at 4 °C overnight. We incubated the cells with Alexa Fluor 594 conjugated goat anti-mouse IgG (A-11005; Invitrogen) or Alexa Fluor 488 conjugated goat anti-rabbit IgG (ab150077; Abcam). Nuclei were counterstained with 4′,6-Diamidino-2-Phenylindole (DAPI). We mounted the cells using mounting medium (Dako, Carpinteria, CA, USA), and captured images using a THUNDER Imager 3D Live Cell & 3D Cell Culture System (Leica Microsystems, Wetzlar, Germany). Using ImageJ software (version 1.52a), the mean fluorescence intensity for each channel in the three distinct regions was quantified. The fluorescence intensities were presented relative to that of the control.

### 2.6. Statistical Analysis

All data are presented as mean ± standard deviation (SD). Statistical significance between groups was determined using an unpaired Student’s *t*-test. One-way analysis of variance (ANOVA) was conducted, followed by Bonferroni post hoc tests, to determine significant differences among multiple groups. Statistical analyses were performed using GraphPad Prism 5 software. Statistical significance was set at *p* < 0.05, * *p* < 0.05, ** *p* < 0.01, and *** *p* < 0.001.

## 3. Results

### 3.1. Genistein Inhibits Cell Proliferation and Induces Apoptosis in CMT-U27 Cells

**Figure 1:**
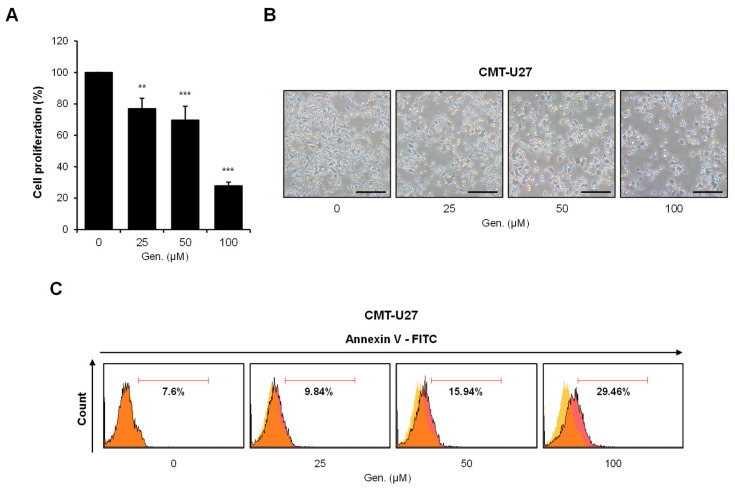
Genistein inhibits cell proliferation and induces apoptosis in CMT-U27 cells. (**A**) CMT-U27 cells were treated with indicated concentrations of genistein for 24 h, and cell viability was assessed using the MTS assay (*n* = 3). Values are presented as mean ± SD. Statistical significance compared to the control group by one-way ANOVA followed by a Bonferroni post hoc test; ** *p* < 0.01, *** *p* < 0.001. (**B**) Cell images show morphological changes in CMT-U27 cells after genistein treatment (*n* = 3). Magnification, 4×. Scale bar, 20 μm. (**C**) Apoptotic cell death was analyzed by flow cytometry with Annexin V-FITC staining (*n* = 3). The yellow gate indicates the control cell population, and the orange gate represents genistein-treated cells exhibiting varying levels of apoptosis. The proportion of Annexin V-positive cells increased in a dose-dependent manner, indicating apoptosis induction by genistein. Gen., Genistein.

Cell viability was measured using the MTS assay after treatment with indicated concentrations of genistein for 24 h to investigate the effect of genistein on CMT-U27 cell proliferation. Genistein significantly inhibited cell proliferation dose-dependently. Compared to that in the control group, cell viability decreased to approximately 77% at 25 μM (*p* < 0.01), 70% at 50 μM (*p* < 0.001), and 28% at 100 μM (*p* < 0.001) (Figure 1A). Morphological observations confirmed the cytotoxic effects of genistein. CMT-U27 cells treated with genistein showed distinct morphological changes, characterized by rounding, detachment from the culture surface, and reduced cell density in a concentration-dependent manner (Figure 1B). Flow cytometric analysis using Annexin V-FITC staining was performed to determine whether genistein-induced cell death occurred via apoptosis. The percentage of Annexin V-positive apoptotic cells increased with genistein treatment, from 7.6% in untreated controls to 9.84% at 25 μM, 15.94% at 50 μM, and 29.46% at 100 μM (Figure 1C).

### 3.2. Genistein Activates the PERK–ATF4–CHOP Pathway in CMT-U27 Cells

**Figure 2:**
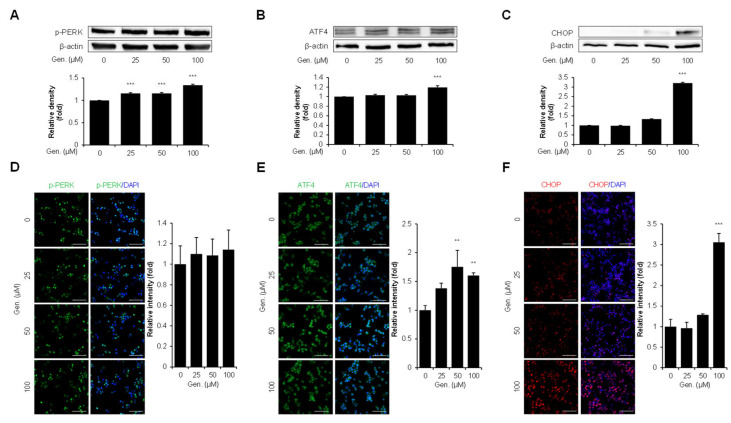
Genistein activates the PERK–ATF4–CHOP pathway in CMT-U27 cells. (**A**–**C**) Western blot images showing the expression levels of p-PERK (**A**), ATF4 (**B**), and CHOP (**C**) in CMT-U27 cells treated with genistein at the indicated concentrations. Protein levels were quantified and normalized to β-actin, and the relative intensities are shown in the bar graphs below (*n* = 3). (**D**–**F**) Immunocytochemistry images showing p-PERK (**D**), ATF4 (**E**), and CHOP (**F**) expression in genistein-treated CMT-U27 cells. The expression levels were detected using specific antibodies (green: p-PERK, ATF4; red: CHOP), and nuclei were counterstained with DAPI (blue). Quantified fluorescence intensities are shown in the adjacent bar graphs (*n* = 5). Values are presented as mean ± SD. Statistical significance compared to the control group by one-way ANOVA followed by a Bonferroni post hoc test; ** *p* < 0.01, *** *p* < 0.001. Gen., Genistein.

Key pathway markers in CMT-U27 cells were evaluated using Western blotting and immunocytochemistry to find out the effect of genistein on the PERK signaling branch of the UPR. Protein analyzation by Western blot demonstrated that p-PERK significantly increased dose-dependently. The relative intensity of p-PERK was significantly increased by 15% at both 25 μM and 50 μM, and by 34% at 100 μM compared to that in the control group (*p* < 0.001) (Figure 2A). Similarly, the expression of ATF4, a downstream transcription factor activated by PERK, was upregulated in response to genistein treatment. ATF4 protein levels increased by approximately 19% at 100 μM compared to that in the untreated group (*p* < 0.001) (Figure 2B). CHOP, a pro-apoptotic ER stress marker, was significantly increased by approximately 220% at 100 μM (*p* < 0.001) (Figure 2C). To confirm these findings, immunocytochemical staining was performed to visualize the expression of p-PERK, ATF4, and CHOP in CMT-U27 cells. In addition, p-PERK was detected in the cytoplasm. There was no significant variation based on statistical evaluation, whereas the fluorescence intensity increased with genistein treatment (Figure 2D). In contrast, the fluorescence intensity of ATF4 increased significantly at 50 μM (75%) and 100 μM (60%) compared to that in the control (*p* < 0.01), which was consistent with the Western blot results (Figure 2E). CHOP expression was markedly upregulated in the nucleus, showing an approximately two-fold increase at 100 μM compared to that in the control (*p* < 0.001) (Figure 2F).

### 3.3. Genistein Promotes CHOP-Mediated Apoptosis via DR5, LC3B–Caspase-8–Caspase-3, and Bax/Bcl-2 Signaling in CMT-U27 Cells

**Figure 3:**
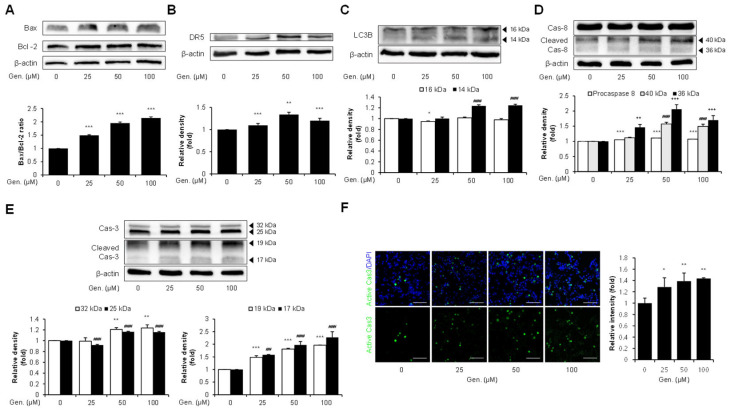
Genistein promotes CHOP-mediated apoptosis via Bax/Bcl-2, and DR5, LC3B–caspase-8–caspase-3 signaling in CMT-U27 cells. (**A**) Western blot images showing the expression level of Bax and Bcl-2 in CMT-U27 cells treated with genistein at the indicated concentration. The Bax/Bcl-2 ratio was calculated and is shown in the bar graphs below (*n* = 3). (**B**) Expression level of DR5, a CHOP-regulated death receptor, was examined in genistein-treated CMT-U27 cells (*n* = 3). (**C**) The expression of LC3B was evaluated by quantifying the levels of LC3B-I (16 kDa) and LC3B-II (14 kDa) (*n* = 3). (**D**,**E**) Protein levels of full-length and cleaved caspase-8 (**D**) and caspase-3 (**E**) were analyzed to investigate the activation of the extrinsic apoptotic pathway (*n* = 3). (**F**) Immunocytochemistry images showing the expression of active caspase-3 (green) in CMT-U27 cells after genistein treatment. Nuclei were counterstained with DAPI (blue) and quantified fluorescence intensity is shown in the bar graph (*n* = 5). Values are presented as mean ± SD. Statistical significance compared to the control group by one-way ANOVA followed by a Bonferroni post hoc test; * *p* < 0.05, ** *p* < 0.01, *** *p* < 0.001. ## *p* < 0.01, ### *p* < 0.001. Gen., Genistein.

The expression of related markers was examined to determine whether CHOP induction by genistein was associated with downstream apoptotic signaling in CMT-U27 cells. The levels of pro-apoptotic Bax and anti-apoptotic Bcl-2 were measured to assess the involvement of the intrinsic apoptotic pathway. Western blot analysis revealed that genistein treatment upregulated the Bax/Bcl-2 ratio, with 49% at 25 μM, 95% at 50 μM and 114% at 100 μM (*p* < 0.001) (Figure 3A). Additionally, genistein significantly increased the expression of DR5, a CHOP-regulated mediator of ER stress-induced apoptosis. DR5 expression was elevated at all tested concentrations, with a 20% increase observed at 100 μM compared to that in the control group (*p* < 0.001) (Figure 3B). Genistein treatment markedly increased the expression of LC3B. Genistein dose-dependently increased LC3B-II (14 kDa) expression, with levels elevated by 24% at 50 μM and 25% at 100 μM (*p* < 0.001) (Figure 3C). We assessed caspase-8 cleavage based on the proposed link between LC3B and caspase-8 activation during ER stress. Although pro-caspase-8 showed a slight increase across treatment groups, cleaved caspase-8 fragments were significantly elevated in response to genistein treatment. Specifically, the 36 kDa fragment increased by 50% at 25 μM (*p* < 0.01), 106% at 50 μM, and 70% at 100 μM, and the 40 kDa fragment increased by 57% at 50 μM and 49% at 100 μM (*p* < 0.001) (Figure 3D). Subsequently, the activation of caspase-3, a key executioner of caspases, was also observed. The pro-caspase-3 bands showed moderate but significant increases, with the 32 kDa band elevated by 20% at 50 μM and 24% at 100 μM (*p* < 0.01), and the 25 kDa band increased by 16% at both 50 and 100 μM (*p* < 0.001). In addition, cleaved caspase-3 fragments were significantly increased dose-dependently; the 17 kDa fragment was elevated by 58%, 98%, and 127%, and the 19 kDa fragment by 48%, 81%, and 96% at 25, 50, and 100 μM, respectively (*p* < 0.001) (Figure 3E). These results were confirmed using immunocytochemical analyses. Consistent with the Western blotting data, active caspase-3 was prominently detected in the cytoplasm of genistein-treated cells. Fluorescence intensity increased significantly by 28% at 25 μM (*p* < 0.05), 40% at 50 μM, and 44% at 100 μM (*p* < 0.01) (Figure 3F).

### 3.4. Genistein Suppresses the IRE1α–XBP1s Pathway in CMT-U27 Cells

**Figure 4:**
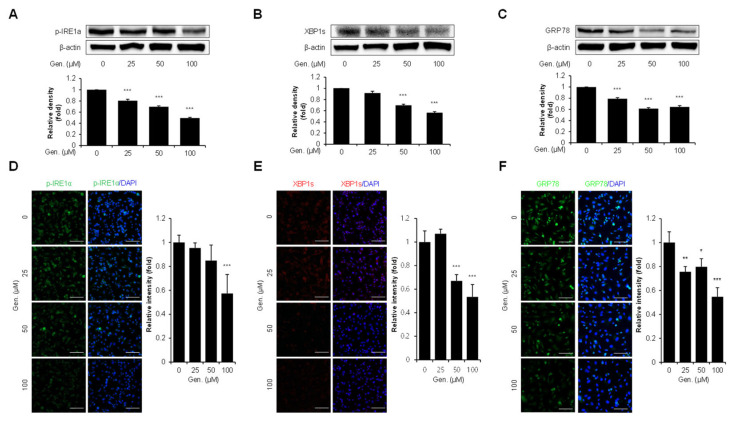
Genistein suppresses the IRE1α–XBP1s pathway in CMT-U27 cells. (**A**–**C**) Western blot images showing the expression levels of p-IRE1α (**A**), XBP1s (**B**), and GRP78 (**C**) in CMT-U27 cells treated with genistein at the indicated concentrations. Protein levels were quantified and normalized to β-actin, and the relative intensities are shown in the bar graphs below (*n* = 3). (**D**–**F**) Immunocytochemistry images showing p-IRE1α (**D**), XBP1s (**E**), and GRP78 (**F**) expression in genistein-treated CMT-U27 cells. The expression levels were detected using specific antibodies (green: p-IRE1α, GRP78; red: XBP1s), and nuclei were counterstained with DAPI (blue). Quantified fluorescence intensities are shown in the adjacent bar graphs (*n* = 5). Values are presented as mean ± SD. Statistical significance compared to the control group by one-way ANOVA followed by a Bonferroni post hoc test; * *p* < 0.05, ** *p* < 0.01, *** *p* < 0.001. Gen., Genistein.

To find out the effect of genistein on the IRE1α branch of the UPR, the expression of p-IRE1α, XBP1s, and GRP78 was evaluated in CMT-U27 cells. Western blot analysis revealed a significant, dose-dependent downregulation of p-IRE1α in genistein-treated cells. Compared with the treated and untreated groups, p-IRE1α densities were decreased by 20% at 25 μM, 30% at 50 μM, and 50% at 100 μM (*p* < 0.001) (Figure 4A). Similarly, XBP1s, a downstream effector of IRE1α, was significantly downregulated by genistein in a concentration-dependent manner. XBP1s expression decreased by 9% at 25 μM, 31% at 50 μM, and 44% at 100 μM compared to that in the control group (*p* < 0.001) (Figure 4B). Genistein treatment significantly downregulated the expression of GRP78, a major ER chaperone and marker of ER stress. GRP78 protein levels were reduced dose-dependently by 20% at 25 μM, 39% at 50 μM, and 36% at 100 μM compared to that in the control group (*p* < 0.001) (Figure 4C). These results were validated using immunocytochemical analyses. The fluorescence intensity of p-IRE1α was significantly reduced by 43% at 100 μM (*p* < 0.001) (Figure 4D) and XBP1s intensity decreased by 33% at 50 μM and 47% at 100 μM (*p* < 0.001) (Figure 4E). Furthermore, GRP78 fluorescence intensity was reduced dose-dependently, with significant decreases of 24% at 25 μM (*p* < 0.01), 20% at 50 μM (*p* < 0.05), and 47% at 100 μM (*p* < 0.001) (Figure 4F).

### 3.5. Genistein Downregulates ERα Expression in CMT-U27 Cells

**Figure 5:**
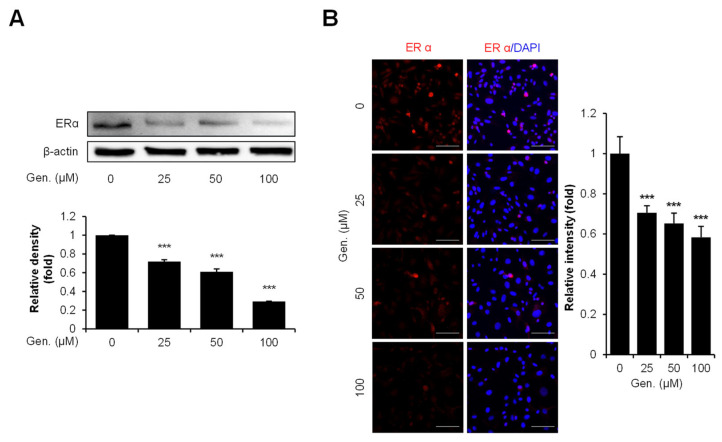
Genistein downregulates ERα expression in CMT-U27 cells. (**A**) Western blot images showing the expression levels of ERα in CMT-U27 cells treated with genistein at the indicated concentrations. Protein levels were normalized to β-actin, and the relative intensities are shown in the bar graph below (*n* = 3). (**B**) Immunocytochemistry images showing ERα expression in genistein-treated CMT-U27 cells. The expression levels were detected using a specific antibody (red: ERα), and nuclei were counterstained with DAPI (blue). Quantified fluorescence intensities are shown in the adjacent bar graph (*n* = 5). Values are presented as mean ± SD. Statistical significance compared to the control group by one-way ANOVA followed by a Bonferroni post hoc test; *** *p* < 0.001. Gen., Genistein.

Western blot and immunocytochemistry analyses were conducted using CMT-U27 cells treated with increasing concentrations of genistein to examine the effect of genistein on ERα expression. Western blot results demonstrated a concentration-dependent decrease in ERα protein levels following genistein treatment. ERα expression was significantly reduced by 29% at 25 μM, 39% at 50 μM, and 70% at 100 μM compared to that in the control group (*p* < 0.001) (Figure 5A). Consistent with these findings, immunocytochemical staining revealed markedly reduced ERα intensity in genistein-treated cells. Quantitative analysis showed a significant reduction in fluorescence intensity by 70% at 25 μM, 65% at 50 μM, and 58% at 100 μM compared to that in the control group (*p* < 0.001) (Figure 5B).

## 4. Discussion

Genistein-induced apoptosis has been linked to increased Bax/Bcl-2 ratio, caspase activation, and ER stress-associated signaling [29]. The UPR is a cellular adaptive mechanism that restores ER homeostasis under stress by enhancing protein-folding capacity or attenuating protein synthesis [30]. However, the UPR shifts from promoting cell survival to activating apoptosis under prolonged or severe ER stress [31]. Since the UPR plays a critical role in cancer cell survival and therapeutic resistance, we investigated whether genistein modulates UPR signaling to exert anticancer effects in CMT-U27 cells.

Our study demonstrated that genistein induces apoptosis in CMT-U27 cells by modulating the UPR pathway. Genistein treatment activated the PERK signaling pathway dose-dependently, with a marked upregulation of CHOP expression. This activation is accompanied by an increased expression of downstream targets, such as DR5 and LC3B. Although LC3B is traditionally recognized as an autophagy marker [32], a study by Paula Lindner et al. reported its role in ER stress-induced apoptosis via caspase-8 activation [33,34]. Consistent with these findings, our data showed that LC3B upregulation was accompanied by increased levels of cleaved caspase-8 and caspase-3, suggesting that LC3B functions as an upstream regulator of caspase-mediated apoptosis under ER stress. Moreover, genistein increased the Bax/Bcl-2 ratio, further supporting its role in activating the intrinsic apoptotic pathway.

**Figure 6:**
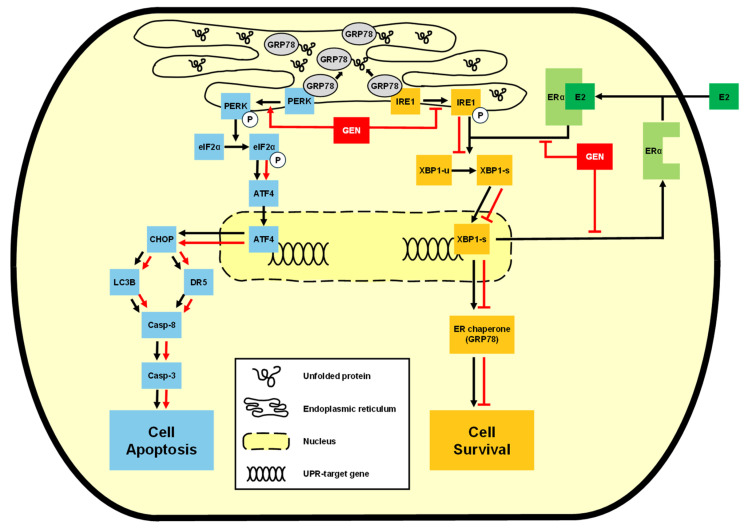
Schematic diagram of the mechanisms of the anticancer effect of genistein.

Our findings also suggested that genistein promoted pro-apoptotic UPR signaling via the PERK axis, while suppressing the pro-survival IRE1α–XBP1s axis at the same time. Western blotting and immunocytochemical analysis confirmed that genistein reduced the expression of p-IRE1α, XBP1s, and GRP78. Given that ERα positively regulates IRE1α expression [35,36,37], and genistein functions as a phytoestrogen, our findings suggest that ERα downregulation by genistein contributes to the suppression of the IRE1α–XBP1s signaling axis. As a result, genistein modulates the UPR by activating the pro-apoptotic PERK–ATF4–CHOP axis and suppressing the pro-survival IRE1α–XBP1s axis in CMT-U27 cells (Figure 6). This dual modulation represents a distinct anticancer mechanism that redirects ER stress-induced UPR signaling from survival to apoptosis. Our study provides evidence for UPR-targeted anticancer effects of genistein in CMTs, establishing a foundation for ER stress modulation as a therapeutic strategy in veterinary oncology. Future investigations expanding to other CMT cell lines and in vivo studies, along with combination therapy approaches using conventional chemotherapeutics, could further validate and enhance the therapeutic potential for CMT treatment.

## 5. Conclusions

In conclusion, this study demonstrated that genistein led to reduced cell viability and increased apoptosis, accompanied by upregulation of the PERK–ATF4–CHOP pathway and downregulation of the IRE1–XBP1 axis. Furthermore, genistein downregulated the expression of ERα, potentially disrupting the positive feedback loop between ERα and IRE1α, thereby contributing to the inhibition of the IRE1α pathway. These findings suggest that genistein induces ER stress-mediated cell death through the activation of pro-apoptotic UPR signaling while suppressing adaptive survival pathways. Taken together, our results highlight the potential of genistein as a modulator of the UPR to treat CMTs.

## Data Availability

The original contributions presented in this study are included in the article/Supplementary Materials. Further inquiries can be directed to the corresponding authors.

## Supplementary Materials

The following supporting information can be downloaded at https://www.mdpi.com/article/10.3390/ani15121717/s1: Figure S1: Immunoblots and densitometry reading/intensity ratio of PERK-ATF4-CHOP in CMT-U27 cells; Figure S2. Immunoblots and densitometry reading/intensity ratio of Bax, Bcl2, DR5 and LC3B in CMT-U27 cells; Figure S3. Immunoblots and densitometry reading/intensity ratio of caspase in CMT-U27 cells; Figure S4. Immunoblots and densitometry reading/intensity ratio of IRE1-XBP1-GRP78 in CMT-U27 cells; Figure S5. Immunoblots and densitometry reading/intensity ratio of ERα in CMT-U27 cells; Figure S6. The half-maximal inhibitory concentration (IC_50_) of genistein in CMT-U27 cells; Figure S7. The gene expression of ATF6 in CMT-U27 cells by RT-PCR.

## Abbreviations

CMT	Canine mammary gland tumor
ER	Endoplasmic reticulum
PERK	Protein kinase-like ER kinase
IRE1α	Inositol-requiring enzyme 1 alpha
ATF6	Activating transcription factor 6
GRP78	Glucose-regulated protein 78
UPR	Unfolded protein response
XBP1	X-box binding protein 1
ESR1	Estrogen receptor 1
ATF4	Activating transcription factor 4
CHOP	C/EBP homologous protein
p-PERK	Phosphorylated PERK
p-IRE1α	Phosphorylated IRE1α
XBP1s	Spliced XBP1
ERα	Estrogen receptor alpha
Bcl2	B-cell lymphoma-2
Bax	Bcl2-associated X
FBS	Fetal bovine serum
MTS	3-(4,5-dimethylthiazol-2-yl)-5-(3-carboxymethoxyphenyl)-2-(4-sulfophenyl)-2H-tetrazolium
PI	Propidium Iodide
DR5	Death receptor 5
LC3B	Microtubule-associated protein 1 light chain 3 beta
HRP	Horse radish peroxidase
PBS	Phosphate-buffered saline
BSA	Bovine serum albumin
DAPI	4′6-Diamidino-2-Phenylindole
SD	Standard deviation
Gen	Genistein
