# Supplementary material for: The Anticancer Effect of Genistein Through Enhancing PERK Signaling and Suppressing the IRE1α-XBP1 Axis in Canine Mammary Gland Tumor Cells
Source: Animals (Basel). 2025 Jun 10;15(12):1717. doi: 10.3390/ani15121717 (PMC12189220; doi:10.3390/ani15121717)
Supplement: Supplementary file 1 [file animals-15-01717-s001.zip › animals-3671492-supplementary.pdf]

animals

a

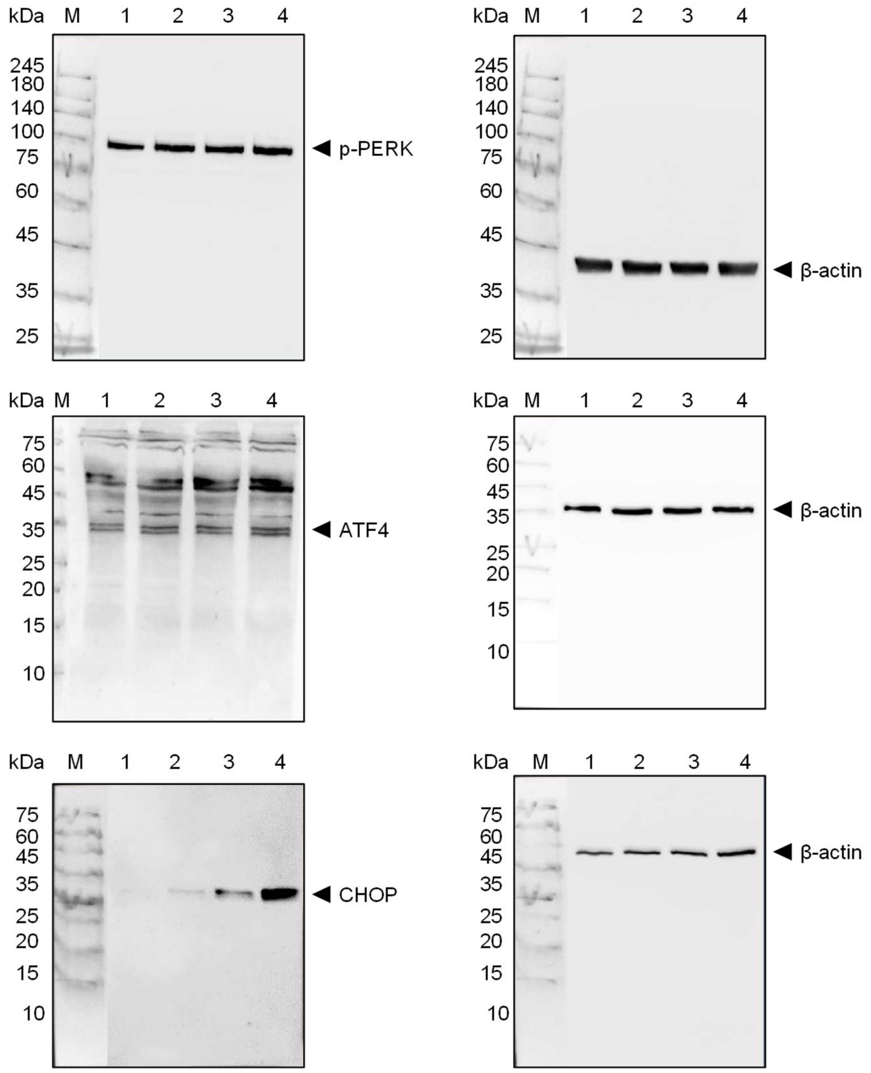

Lane M: Molecular weight markers
Lane 1: Untreated
Lane 2: Genistein (25 μM)
Lane 3: Genistein (50 μM)
Lane 4: Genistein (100 μM)

b

Genistein (μM)	p-PERK/β-actin	ATF4/β-actin	CHOP/β-actin
**0**	1.000 ± 0.004	1.000 ± 0.069	1.000 ± 0.060
**25**	1.154 ± 0.013	1.030 ± 0.052	0.982 ± 0.065
**50**	1.155 ± 0.015	1.028 ± 0.054	1.329 ± 0.058
**100**	1.341 ± 0.016	1.193 ± 0.048	3.201 ± 0.069

Figure S1. Immunoblots and densitometry reading/intensity ratio of PERK-ATF4-CHOP in CMT-U27 cells. CMT-U27 cells were treated with 0, 25, 50, and 100 μM of genistein for 18 h. The protein expression of p-PERK (125 kDa), ATF4 (39 kDa), CHOP (29 kDa) was analyzed by (a) western blots and (b) densitometry reading/intesntiy ratio. The band intensity was normalized to the corresponding β-actin value. PERK, protein kinase R-like endoplasmic reticulum kinase; p-PERK, phosphorylated PERK; ATF4, activating transcription factor 4; CHOP, C/EBP homologous protein.

a

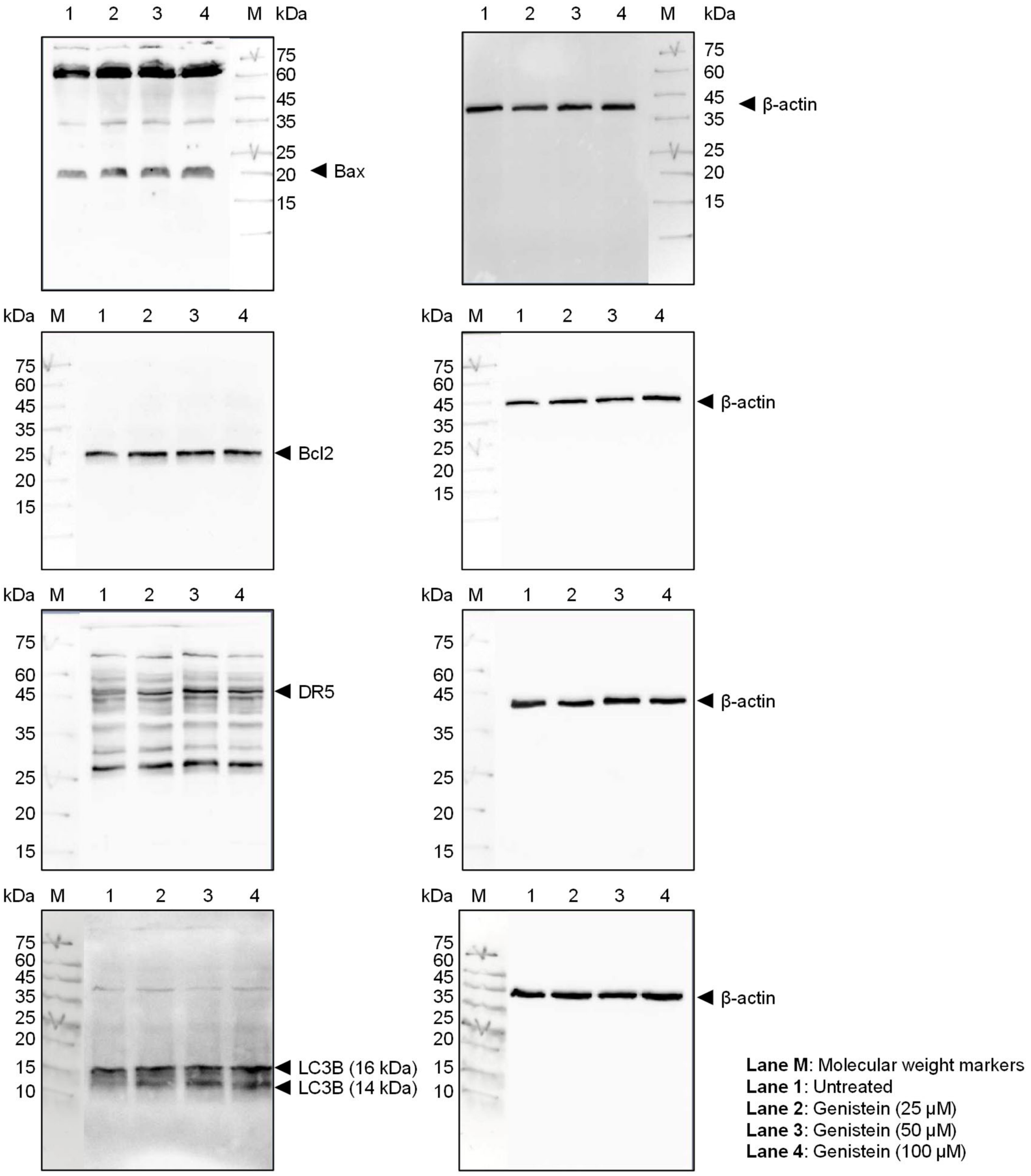

b

Genistein (μM)	Bax/β-actin	Bcl2/β-actin	DR5/β-actin	LC3B(16 kDa)/β-actin	LC3B(14 kDa)/β-actin
**0**	1.000 ± 0.004	1.000 ± 0.011	1.000 ± 0.031	1.000 ± 0.009	1.000 ± 0.008
**25**	1.531 ± 0.008	1.025 ± 0.004	1.095 ± 0.010	0.908 ± 0.041	1.009 ± 0.004
**50**	2.092 ± 0.007	1.071 ± 0.005	1.335 ± 0.014	1.026 ± 0.054	1.202 ± 0.013
**100**	1.649 ± 0.007	0.769 ± 0.001	1.200 ± 0.021	1.047 ± 0.016	1.135 ± 0.008

Figure S2. Immunoblots and densitometry reading/intensity ratio of Bax, Bcl2, DR5 and LC3B in CMT-U27 cells. CMT-U27 cells were treated with 0, 25, 50, and 100 μM of genistein for 18 h. The protein expression of Bax (20 kDa), Bcl2 (26kDa), DR5 (48 kDa), LC3B-I (16 kDa), LC3B-II (14 kDa) was analyzed by (a) western blots and (b) densitometry reading/intesntiy ratio. The band intensity was normalized to the corresponding β-actin value. Bax, Bcl-2-associated X; Bcl2, B-cell lymphoma-2; DR5, death receptor 5; LC3B, microtubule-associated protein 1 light chain 3 beta.

 https://doi.org/10.3390/xxxxx

a

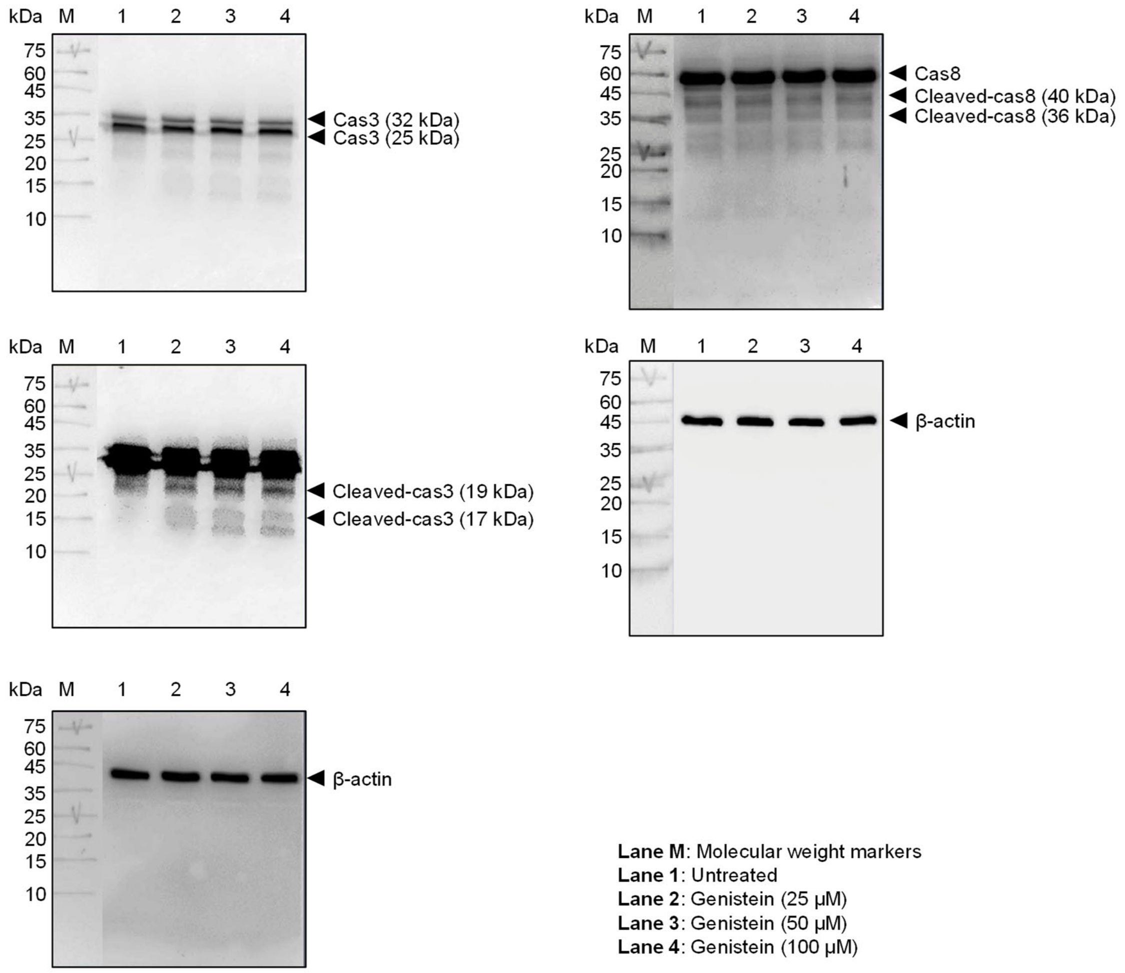

b

Genistein (μM)	Cas3 (32 kDa) /β-actin	Cas3 (25 kDa) /β-actin	Cleaved-cas3 (19 kDa) /β-actin	Cleaved-cas3 (17 kDa) /β-actin
0	1.000 ± 0.035	1.000 ± 0.008	1.000 ± 0.017	1.000 ± 0.050
25	0.989 ± 0.031	0.919 ± 0.006	1.481 ± 0.042	1.581 ± 0.071
50	1.207 ± 0.012	1.164 ± 0.002	1.808 ± 0.003	1.974 ± 0.036
100	1.234 ± 0.016	1.159 ± 0.003	1.963 ± 0.031	2.267 ± 0.112

Genistein (μM)	Cas8/β-actin	Cleaved-cas8 (40 kDa)/β-actin	Cleaved-cas8 (36 kDa)/β-actin
0	1.000 ± 0.002	1.000 ± 0.027	1.000 ± 0.060
25	1.051 ± 0.002	1.117 ± 0.013	1.462 ± 0.011
50	1.108 ± 0.003	1.572 ± 0.015	2.054 ± 0.025
100	1.074 ± 0.002	1.492 ± 0.032	1.696 ± 0.046

Figure S3. Immunoblots and densitometry reading/intensity ratio of caspase in CMT-U27 cells. CMT-U27 cells were treated with 0, 25, 50, and 100 μM of genistein for 18 h. The protein expression of cas3 (32 kDa, 25 kDa), cleaved-cas3 (19 kDa, 17 kDa), cas8 (55 kDa), cleaved-cas8 (40 kDa, 36 kDa) was analyzed by (a) western blots and (b) densitometry reading/intesntiy ratio. The band intensity was normalized to the corresponding β-actin value. Cas3, caspase3; Cas8, caspase8.

a

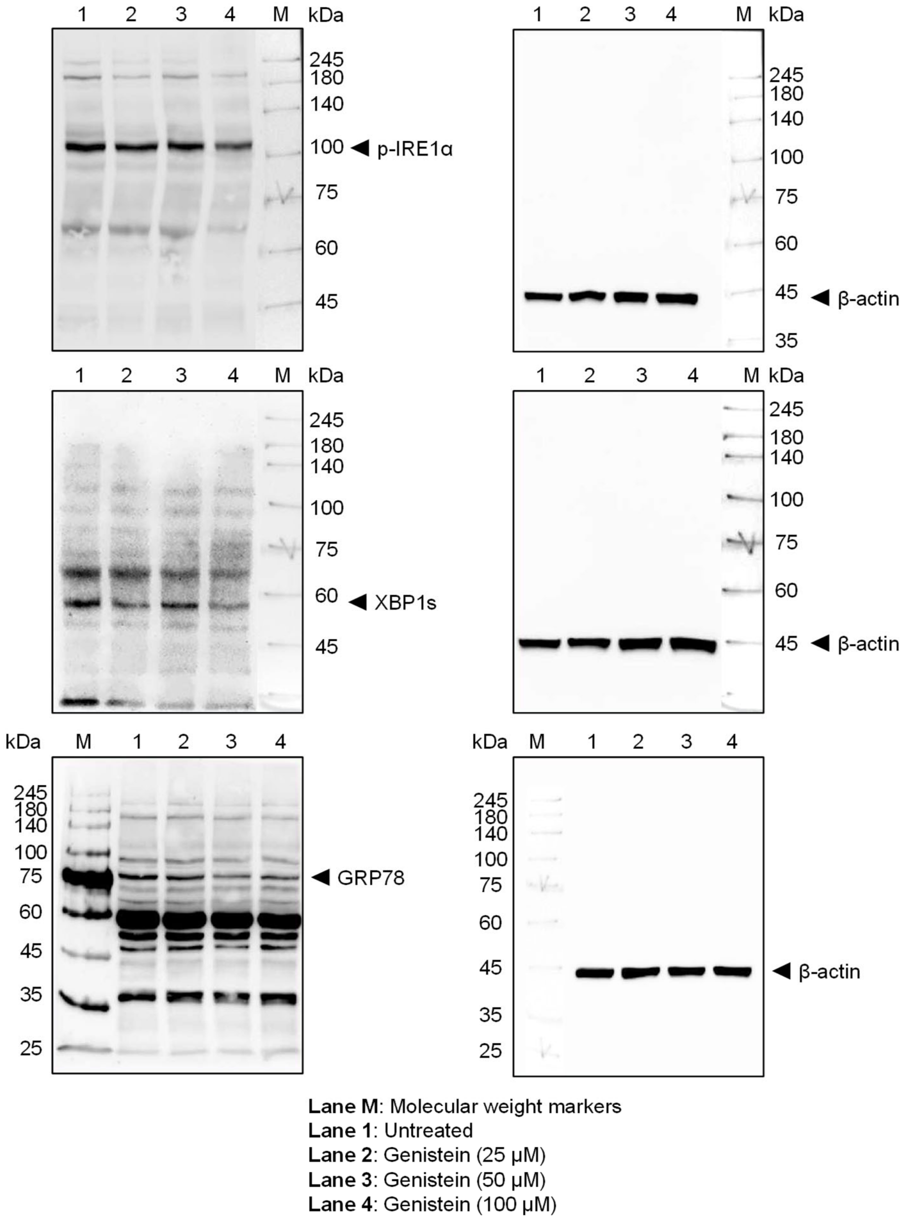

b

Genistein (μM)	p-IRE1α/β-actin	XBP1s/β-actin	GRP78/β-actin
**0**	1.000 ± 0.013	1.000 ± 0.056	1.000 ± 0.004
**25**	0.870 ± 0.018	0.911 ± 0.028	0.791 ± 0.016
**50**	0.849 ± 0.027	0.694 ± 0.030	0.613 ± 0.019
**100**	0.713 ± 0.030	0.560 ± 0.032	0.643 ± 0.021

Figure S4. Immunoblots and densitometry reading/intensity ratio of IRE1-XBP1-GRP78 in CMT-U27 cells. CMT-U27 cells were treated with 0, 25, 50, and 100 μM of genistein for 18 h. The protein expression of p-IRE1α (110 kDa), XBP1s (60 kDa), GRP78 (78 kDa) was analyzed by (a) western blots and (b) densitometry reading/intesntiy ratio. The band intensity was normalized to the corresponding β-actin value. IRE1α, inositol-requiring enzyme 1 alpha; p-IRE1α, phosphorylated IRE1α; XBP1, X-box binding protein 1; XBP1s, spliced XBP1; GRP78, glucose-regulated protein 78.

a

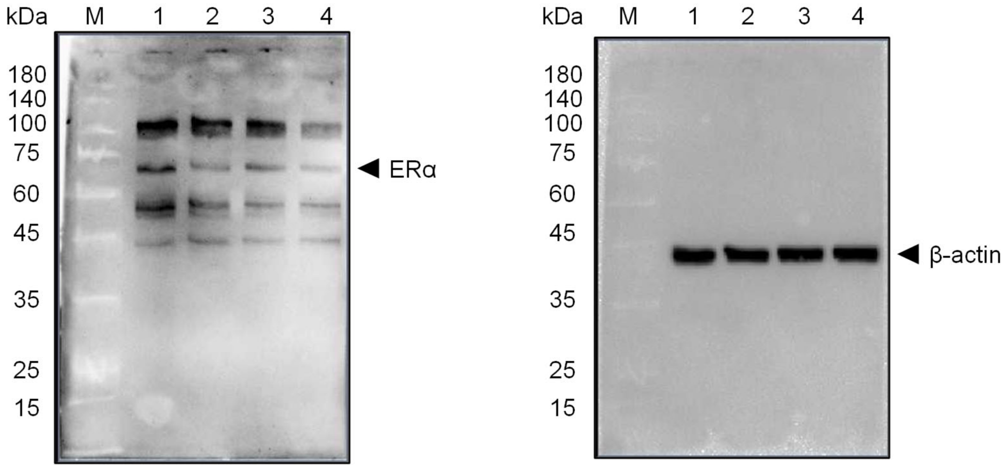

Lane M: Molecular weight markers
Lane 1: Untreated
Lane 2: Genistein (25 μM)
Lane 3: Genistein (50 μM)
Lane 4: Genistein (100 μM)

b

Genistein (μM)	ERα/β-actin
**0**	1.000 ± 0.019
**25**	0.719 ± 0.006
**50**	0.610 ± 0.017
**100**	0.293 ± 0.003

Figure S5. Immunoblots and densitometry reading/intensity ratio of ER*α* in CMT-U27 cells. CMT-U27 cells were treated with 0, 25, 50, and 100 μM of genistein for 18 h. The protein expression of ER*α* (55 kDa) was analyzed by (a) western blots and (b) densitometry reading/intesntiy ratio. The band intensity was normalized to the corresponding β-actin value. ER*α*, estrogen receptor *α*.

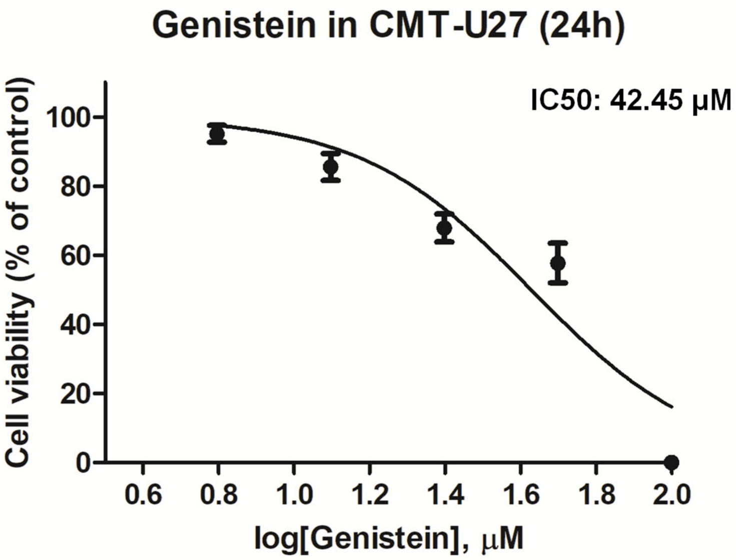

Figure S6. The half-maximal inhibitory concentration (IC_{50}) of genistein in CMT-U27 cells. The figures show the 50% inhibitory concentrations (IC50) of the genistein in CMT-U27 cells. Cells were treated with indicated concentrations for 24 h and cell viability was determined using the MTS assay. The IC50 value was calculated using nonlinear regression analysis (n=3).

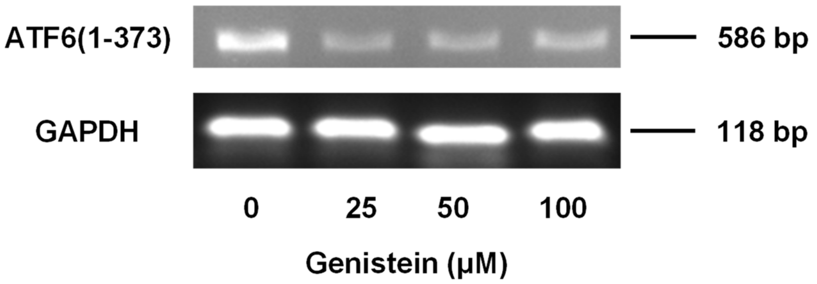

Table 1. Oligonucleotide primers used for gene expression analysis.

Gene	Primer sequence	Size (bp)
ATF6(1-373)	(F) 5′-GCCTTTATTGCTTCCAGCAG-3′<br>(R) 5′-TGAGACAGCAAAACCGTCTG-3′	586
GAPDH	(F) 5′-GGAGAAAGCTGCCAAATATGACG-3′<br>(R) 5′-ACTGTTGAAGTCACAGGAGACC-3′	118

Figure S7. The gene expression of ATF6 in CMT-U27 cells by RT-PCR. CMT-U27 cells were treated with 0, 25, 50, and 100 μM of genistein for 18 h. The gene expression of ATF6 was analyzed by RT-PCR with indicated primer.
